# Combined Efficacy of Cediranib and Quinacrine in Glioma Is Enhanced by Hypoxia and Causally Linked to Autophagic Vacuole Accumulation

**DOI:** 10.1371/journal.pone.0114110

**Published:** 2014-12-09

**Authors:** Merryl R. Lobo, Xiaoyan Wang, G. Yancey Gillespie, Randall L. Woltjer, Martin M. Pike

**Affiliations:** 1 Advanced Imaging Research Center, Oregon Health and Science University, Portland, Oregon, United States of America; 2 Department of Biomedical Engineering, Oregon Health and Science University, Portland, Oregon, United States of America; 3 Department of Pathology, Oregon Health and Science University, Portland, Oregon, United States of America; 4 Department of Molecular and Medical Genetics at Oregon Health and Science University, Portland, Oregon, United States of America; 5 Department of Neurosurgery, University of Alabama at Birmingham, Birmingham, Alabama, United States of America; Swedish Medical Center, United States of America

## Abstract

We have previously reported that the *in vivo* anti-glioma efficacy of the anti-angiogenic receptor tyrosine kinase inhibitor cediranib is substantially enhanced via combination with the late-stage autophagy inhibitor quinacrine. The current study investigates the role of hypoxia and autophagy in combined cediranib/quinacrine efficacy. EF5 immunostaining revealed a prevalence of hypoxia in mouse intracranial 4C8 glioma, consistent with high-grade glioma. MTS cell viability assays using 4C8 glioma cells revealed that hypoxia potentiated the efficacy of combined cediranib/quinacrine: cell viability reductions induced by 1 µM cediranib +2.5 µM quinacrine were 78±7% (hypoxia) vs. 31±3% (normoxia), p<0.05. Apoptosis was markedly increased for cediranib/quinacrine/hypoxia versus all other groups. Autophagic vacuole biomarker LC3-II increased robustly in response to cediranib, quinacrine, or hypoxia. Combined cediranib/quinacrine increased LC3-II further, with the largest increases occurring with combined cediranib/quinacrine/hypoxia. Early stage autophagy inhibitor 3-MA prevented LC3-II accumulation with combined cediranib/quinacrine/hypoxia and substantially attenuated the associated reduction in cell viability. Combined efficacy of cediranib with bafilomycin A1, another late-stage autophagy inhibitor, was additive but lacked substantial potentiation by hypoxia. Substantially lower LC3-II accumulation was observed with bafilomycin A1 in comparison to quinacrine. Cediranib and quinacrine each strongly inhibited Akt phosphoryation, while bafilomycin A1 had no effect. Our results provide compelling evidence that autophagic vacuole accumulation plays a causal role in the anti-glioma cytotoxic efficacy of combined cediranib/quinacrine. Such accumulation is likely related to stimulation of autophagosome induction by hypoxia, which is prevalent in the glioma tumor microenvironment, as well as Akt signaling inhibition from both cediranib and quinacrine. Quinacrine's unique ability to inhibit both Akt and autophagic vacuole degradation may enhance its ability to drive cytotoxic autophagic vacuole accumulation. These findings provide a rationale for a clinical evaluation of combined cediranib/quinacrine therapy for malignant glioma.

## Introduction

Malignant gliomas are the most frequently occurring primary malignant brain tumors in adults. Glioblastoma multiforme (GBM), the most common malignant glioma, represents their most severe manifestation with an average survival of 15 months, despite improvements in diagnosis and treatment [1], [2]. Standard-of-care treatment involves surgical resection, radiotherapy and concomitant and adjuvant chemotherapy with temozolomide. More recently, a deeper understanding of the molecular pathology of glioblastoma in patients has promoted the exploration of a more targeted therapeutic approach. Growth factor receptor pathways, such as epidermal growth factor receptor (EGFR), platelet derived growth factor receptor (PDGFR), vascular endothelial growth factor (VEGFR), and others can be excessively activated due to overexpression or mutation of the receptors or ligands [3], [4], [5]. Such aberrant growth factor signaling can drive glioma growth by promoting proliferation, apoptotic resistance, invasion, angiogenesis, and other processes. Thus receptor tyrosine kinase (RTK) inhibitors have been a major focus of drug development. Relevant RTK inhibitors have been tested in a number of clinical studies, and although these agents have shown significant clinical success in many types of tumors, they have not been able to effectively improve clinical survival for GBM [3], [4], [5]. Reasons for the lack of efficacy may include the development of resistance mechanisms in glioblastomas that could induce tolerance to treatment [5], [6]. Autophagy is an essential cellular recycling mechanism that has been shown to exert protective effects in tumors in response to hypoxic/nutrient stress as well as treatment with various anticancer agents [7], [8], [9], [10]. During autophagy, cytoplasmic components are sequestered into double-membrane vesicles called autophagosomes that fuse with cellular lysosomes, thus degrading the contents to provide a temporary source of biosynthetic substrates and energy. A number of studies have shown that a late stage inhibition of autophagy results in an accumulation of autophagic vacuoles (a generic term for all autophagic structures) in the cytoplasm, leading to tumor cell death via either apoptosis dependent or independent mechanisms [9], [11], [12], [13], [14], [15], [16], [17], [18]. Thus, in the context of treatment-induced increased autophagic flux in tumor cells, an appropriate modulation of this process could enhance the efficacy of the anticancer treatment.

We have previously reported that cediranib, a RTK inhibitor targeting VEGF and PDGF receptor signaling was largely unable to provide an effective therapeutic benefit in an intracranial mouse glioma model [19], [20]. However, the combination of cediranib with the late stage autophagy inhibitor quinacrine showed significantly enhanced anti-tumor and anti-angiogenic effects. The study revealed that combined treatment substantially slowed tumor growth and markedly increased both tumor necrosis and mouse survival in comparison to no treatment or treatment with either drug alone. Additionally, perfusion MRI revealed a potent devascularization in tumors with the combined treatment, with significant and sustained reductions in mean tumor cerebral blood flow, volume and vascular permeability. We probed the levels of autophagy marker LC3II (microtubule-associated protein 1 light chain 3) in tumor cell cultures, and showed that cediranib directly induced autophagic flux in glioma cells, an effect which was further increased under hypoxic conditions. Because combined cediranib/quinacrine under hypoxic conditions induced maximal levels of autophagic vacuole accumulation and apoptosis, we hypothesized that autophagic vacuole accumulation played a causal role in the synergistic effect. In the present study, we carefully investigated the relation between hypoxia and the efficacy of the single and combined therapeutic agents. The causality of autophagic vacuole accumulation in the therapeutic efficacy was directly tested. In addition, we investigated the underlying mechanisms by which the two agents modulate the autophagic pathway. We determined that autophagic vacuole accumulation played a key role in cediranib/quinacrine treatment efficacy, and that hypoxia potentiated the synergistic efficacy of the combination. The experiments also revealed quinacrine to be a unique and effective therapeutic agent, which may both stimulate autophagsome induction while inhibiting autophagic vacuole degradation.

## Materials and Methods

### Ethics Statement

Mouse studies were conducted with the approval of the Oregon Health and Science University Institutional Animal Care and Use Committee (protocol #IS00001409) and under the supervision of the OHSU department of Comparative Medicine.

### Cell culture and Tumor Inoculation

The 4C8 mouse glioma cells were provided by Dr. G. Yancey Gillespie, University of Alabama at Birmingham.. The 4C8 cells were originally obtained from Dr. Charissa Dyer, at the Children's Hospital of Philadelphia, Philadelphia, Pennsylvania, and are derived from a clone of a MOCH-1 tumor that arose spontaneously in the brain of a B6D2F1 mouse transgenic for myelin basic protein promoter–driven *c-neu* [21]. The 4C8 cells were grown in Dulbecco's modified Eagle's medium/Ham's F-12 50/50 Mix (Invitrogen, Carlsbad, California), supplemented with 7% fetal bovine serum (FBS) (Hyclone, Logan, Utah) and 1% L-glutamine (Sigma-Aldrich, St. Louis, Missouri). To simulate hypoxic conditions of the tumor, cells were incubated in the BioSpherix hypoxic chamber (BioSpherix, Ltd., Lacona, NY) at 0.5% O_2_. Female C57BL/6× DBA/2 F_1_ hybrid mice (B6D2F1) were purchased from Charles River Laboratories (Wilmington, MA). Brain tumors were induced by the intracerebral injection of 0.5–1×10^6^ 4C8 cells, suspended in DMEM/F12 (5–10 µl) using a stereotaxic frame as previously described [22]. Cediranib (Selleck Chemicals) was prepared initially as a 125 mmol/L stock solution in DMSO and diluted into phosphate buffered saline (10 mmol/L) for further dilution into the relevant assay media. Quinacrine, and bafilomycin A1 were purchased from Sigma-Aldrich.

### Immunohistochemistry

For the assessment of hypoxic regions within the tumors, mice were injected intravenously with the 2-nitroimidazole hypoxia marker EF5 (30 mg/kg, provided by Dr. Cameron J. Koch and the National Cancer Institute/Cancer Therapy Evaluation Program), 3 hours prior to sacrifice. Mouse brains were removed, placed in a cryomold with OCT on dry ice, and stored at −80°C. Sections (10 µm) were mounted on slides in a cryostat, fixed in 4% paraformaldehyde for 60 min, rinsed, and blocked overnight at 4°C. Sections were rinsed and incubated with Alexa 488 conjugated EF5 antibody (provided by Dr Cameron J. Koch) for 6 hours at 4°C while covered with aluminum foil. Sections were subsequently rinsed and mounted with Prolong Gold Anti-fade reagent with DAPI (Life Technologies).

### Cell viability assays

Cells were plated in 96-well plates at a density of 3000 cells/well and were exposed to a range of treatment conditions. To assess cell viability in response to treatment, a mixture of 2 ml of MTS solution (CellTiter 96 AQueous MTS Reagent, Promega) and 100 µl of phenazine methosulfate solution (PMS, Sigma-Aldrich) was added to cells in 96 well plates (20 µL/well) and left for 1 hour at 37°C. A negative control of MTS/PMS stock solution and medium alone was also included. The absorbance was measured using ELX800 Micro Plate Reader (Bio-Tek Instruments, Inc., Winooski, VT) at 490 nm.

### Western blot analysis

Cells were plated at density of 4.8×10^4^ cells per well in 6-well plates and treated with vehicle or drugs. After the indicated time of treatment, cells were lysed by brief sonication in whole-cell lysis buffer (62.5 mM TRIS, 10% glycerol, 1% SDS, pH 6.8), and cellular proteins were collected in the supernatant fraction after centrifugation at 14,000 g for 10 minutes. Proteins were reduced by heating at 100°C for 5 minutes in 50 mmol/L DTT with 0.05 bromphenol blue, electrophoresed in 4–20% Tris-HCl gels, and transferred onto nitrocellulose using the NuPage electrophoresis system (Invitrogen, Carlsbad, CA). The membranes were probed with antibodies to LC3 and cleaved caspase 3 (Cell Signaling, Danvers, MA USA) and actin (Santa Cruz Biotechnology Company, Santa Cruz, CA). Membranes were treated with appropriate secondary antibody, exposed to enhanced chemiluminescence solutions (Invitrogen, Carlsbad, CA) and visualized using the Molecular Imager Gel Documentation System.

### Microscopy for RFP-LC3 expression

Cells were tranduced with lentiviral particles (*EMD Millipore*, Billerica, MA) to stably express the RFP-LC3 fusion protein. Cells were then treated with combined cediranib/quinacrine at the concentrations indicated in the figure legends, for 24 hours. RFP-LC3 expressing cells were then visualized using an EVOS fl fluorescence microscope (AMG).

### Statistical analysis

Values are expressed as mean ± SEM. Student's t test (*NCSS Statistical Software*, (Kaysville, UT) was employed to test for statistical significance.

## Results

### The presence of hypoxia in 4C8 glioma *in vivo*

**Figure 1:**
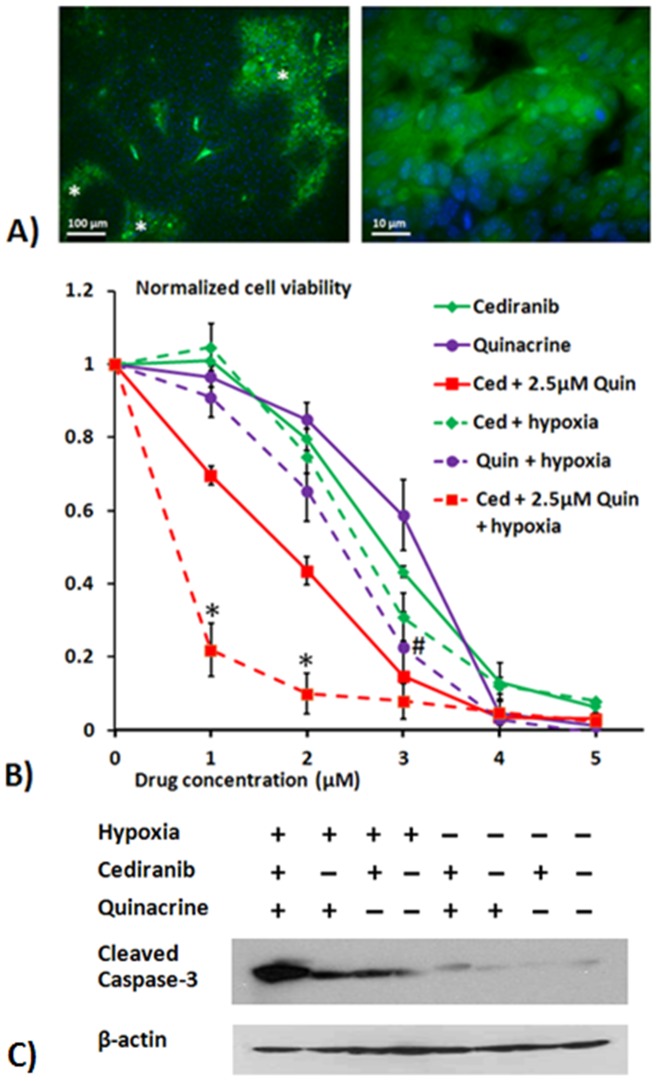
Hypoxia's presence *in vivo* and its effect on cediranib/quinacrine efficacy *in vitro*. A) Representative tumor sections showing immunohistochemical detection of hypoxic regions in 4C8 glioma, shown under low (left panel) and high (right panel) magnification. Sections were obtained from 3 mice, 2 weeks post initiation of tumor growth. Hypoxic cells were identified using Alexa488 labeled anti-EF5 antibody (green). Nuclei were stained with DAPI (blue). Regions marked with * denote positive staining for hypoxia in the low magnification image. B) Results from MTS cell viability assays showing mean 4C8 cell viability in response to treatment with cediranib and/or quinacrine, normalized to untreated samples. Assays were performed after cells were exposed to treatment for 72 hours, under normoxic or hypoxic (0.5% O_2_) conditions. Results are mean ± SEM of three independent experiments, performed in triplicate. Significant differences between tumor cell viability results under hypoxic and normoxic are denoted by # (p<0.05) for single agent quinacrine treatment and * (p<0.05) for cediranib/quinacrine combination treatment. C) Representative cleaved caspase-3 western blots obtained from 4C8 cells grown under normoxic or hypoxic conditions for 72 hrs while untreated or exposed to cediranib (2 µM), quinacrine (2 µM), or combined cediranib/quinacrine.

Despite its vascularity, malignant glioma is a heterogeneous tumor with largely inefficient vasculature, characterized by dilated, tortuous and highly permeable vessels, resulting in the presence of locally hypoxic regions [23], [24]. Although evidence suggests that a state of chronic hypoxia exists in most tumors, the effect of hypoxia on treatment efficacy of anti-cancer therapeutic agents has not been extensively studied [1], [8], [10], [24], [25]. Because of the potentially important role of hypoxia in treatment efficacy, and to help link the observations of the current study to our previous *in vivo* study of Cediranib/quincrine efficacy 4C8 glioma, we employed EF5 immunostaining to determine whether intracranial 4C8 mouse glioma exhibits regions of hypoxia, congruent with human glioma. The syngeneic intracranial 4C8 mouse glioma model employs immunocompetent wild-type F1 hybrid mice and is characterized by aggressive tumor growth with robust neovasculature and core necrosis [19], [21], [22], [26]. Fig. 1A indicates representative EF5 immunostained sections of intracranial 4C8 glioma, obtained two weeks after tumor growth initiation. The low magnification image reveals that substantial regions of positive EF5 staining, indicating hypoxia, are present within the tissue. Furthermore as EF5 hypoxia detection occurs only within viable cells, it may exclude hypoxic regions which are largely necrotic. The high magnification image displays EF5 stained hypoxic cells within the 4C8 glioma, co-stained with DAPI nuclear stain (blue). These data support the concept that hypoxia is a key aspect of the glioma microenvironment which requires evaluation in terms of its effects on therapeutic efficacy.

### Anti-glioma efficacy with combined cediranib and quinacrine is potentiated by hypoxia

Fig. 1B shows the effect of cediranib, quinacrine and their combination on tumor cell viability using MTS assays, under both normoxic and hypoxic conditions, and normalized to their respective (hypoxic or normoxic) untreated controls. We employed 0.5% O_2_ which is within the range of “moderate hypoxia” (0.1–1%) in terms of tumor oxygenation [27]. Both agents induced dose dependant reductions in cell viability at micromolar concentrations. While hypoxia did not affect cediranib cytotoxicity, it substantially increased that of quinacrine, which decreased tumor cell viability in the presence of hypoxia significantly more than when under normoxic conditions. Combined cediranib/quinacrine treatment decreased tumor cell viability in an approximately additive fashion under normoxic conditions. Importantly, the hypoxic environment significantly potentiated the combined efficacy: 1 µM cediranib with 2.5 µM quinacrine decreased tumor cell viability by 31±3% under normoxic conditions and 78±7% under hypoxic conditions, an additional cell viability reduction of 47%. In contrast, while single agent quinacrine administration was also more efficacious under hypoxic conditions, the analogous additional cell viability reduction under hypoxic conditions with 2.5 µM quinacrine alone was 28% (interpolated). The data suggest that while hypoxia potentiates quinacrine efficacy, the potentiation is amplified with combined cediranib/quinacine. The data further suggest that in contrast to that observed under normoxic conditions, the 80% reduction of cell viability with combined cediranib/quinacrine (1 µM/2.5 µM) under hypoxic conditions, is a greater than additive effect. In comparison, single agent treatment at those concentrations produces no cell viability reduction with cediranib and <60% with quinacrine. It is important to note that Fig. 1B cell viability is normalized to the controls obtained under the respective oxygenation conditions in order to appropriately compare drug efficacy. Direct comparisons of the controls indicated that hypoxic control viability was 0.51±0.05 of that of normoxic at the end of the 72 hour incubation period. Hence, in addition to increasing the efficacy of quinacrine and combined cediranib/quinacrine, hypoxia also independently reduces glioma cell viability. We next assessed whether the combinatorial effects on cell viability were associated with increased apoptotic induction by probing for levels of caspase-3 activation, which is critical to apoptotic cell death. 4C8 glioma cells were exposed to cediranib and quinacrine individually and in combination, under normoxic and hypoxic conditions. Fig. 1C indicates that minimal caspase-3 cleavage was detected in untreated and single agent treated normoxic treatment groups, suggesting that the decrease in cell viability observed in Fig. 1B at comparable drug concentrations is likely to include effects on cell proliferation and induction of non-apoptotic cell death. Increased caspase-3 cleavage was observed with combined cediranib/quinacrine under normoxic conditions. Caspase-3 cleavage increased further with untreated hypoxia, suggesting an induction of apoptosis with hypoxia, consistent both with literature reports and our measured reduction of control cell viability with hypoxia [28]. Further increases in caspase-3 cleavage were observed with single agent cediranib or quinacrine treatment under hypoxic conditions. Clearly the most prominent effect observed however, was the markedly increased caspase-3 cleavage with combined cediranib/quinacrine under hypoxic conditions, consistent with a previous report from our laboratory [19]. These data indicate that hypoxia strongly potentiates the induction of apoptosis by combined cediranib/quinacrine treatment. This observation is consistent with the cell viability data (Fig. 1B), thus providing two independent measures indicating that the most potent anti-glioma effects occur with combined cediranib/quinacrine under hypoxic conditions.

### Cediranib and quinacrine modulate the autophagic pathway in glioma cells

**Figure 2:**
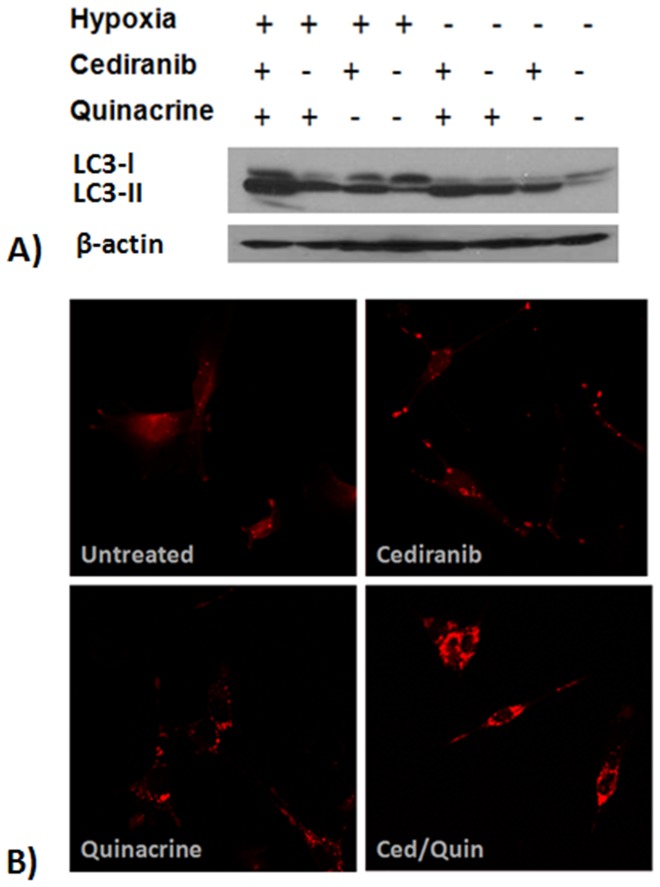
The effect of hypoxia and cediranib/quinacrine on LC3. A) Representative LC3 western blots obtained from 4C8 cells grown under normoxic or hypoxic (0.5% O_2_) conditions for 10 hrs while untreated or exposed to cediranib (3 µM), quinacrine (0.8 µM), or combined cediranib/quinacrine. B) RFP-LC3 expressing 4C8 cells were visualized using Zeiss LSM 780 laser scanning confocal microscope after incubation under hypoxic conditions for 24 hr while untreated or exposed to cediranib (2.5 µM), quinacrine (2.5 µM), or combined cediranib/quinacrine.

During the process of autophagy, autophagosome formation involves conjugation of the cytosolic form of LC3 (LC3-I) to phosphatidylethanolamine to form LC3-II, which becomes recruited to the autophagosome membranes. LC3-II has been proposed to have important functions in the sequestration of ubiquitinated substrates targeted for degradation through its interaction with p62/SQSTM1 [29]. An increase in LC3-II levels in response to treatment can be a result of an increase in autophagic vacuole formation and/or inhibition of autophagic vacuole degradation. To assess the effects of cediranib on autophagic induction in hypoxic and normoxic glioma cells, we exposed 4C8 cells to cediranib treatment under both conditions and probed for levels of LC3-II using western blotting. We implemented analogous experiments to study the effects of quinacrine treatment as well as the combination of the two agents on LC3-II. In these experiments, we employed a submaximal concentration of quinacrine (0.8 µM), to retain sensitivity of the LC3 western blots to alterations in autophagic stimulation, so as to best determine how the combined agents modulate the autophagic process. Fig. 2A shows that hypoxia alone increased LC3-II levels in glioma cells, in agreement with the known stimulatory effect of hypoxia on autophagic flux [10], [29]. Quinacrine treatment also resulted in increased LC3-II levels, in agreement with its action as a late stage autophagy inhibitor [17], [18], [30]. Importantly, substantially increased levels of LC3-II were also observed with cediranib, in comparison to untreated. Further increases in LC3-II accumulation were observed with combined cediranib/quinacrine under normoxic conditions, in comparison to normoxic single agent administration of the agents. Maximum LC3-II accumulation occurred with the combination treatment, under hypoxic conditions, consistent with a previous report from our laboratory [19]. To further investigate autophagic vacuole accumulation, 4C8 cells were transfected with RFP-LC3 and visualized using a Zeiss LSM 780 laser scanning confocal microscope after incubation under hypoxic conditions for 24 hours and exposed to single agent and combined treatment. Fig. 2B shows a largely diffuse cytosolic distribution of the LC3-II protein in untreated cells with relatively few punctate structures, whereas there is an increase in the number of punctae, representing autophagic vacuoles, in response to single agent treatment with either cediranib or quinacrine. The presence of these vacuoles was much higher in response to combination treatment. These data suggest that cediranib and hypoxia stimulate autophagy, which in combination with quinacrine, induces an excessive accumulation of autophagic vacuoles. Although direct comparisons between the assessments of apoptosis, cell viability, and LC3-II accumulation are complicated by the different drug concentrations employed, similarities are evident. At submaximal quinacrine concentrations (Figs. 1B and 2A), combination with Cediranib and hypoxia clearly induced a maximal response in terms of both cell viability reduction and LC3-II accumulation. Analogously, a maximal response of AV accumulation and apoptosis induction (Figs. IC and 2B) was observed when cediranib and hypoxia were combined with quinacrine values approximating half-maximal inhibition. Because combined cediranib/quinacrine/hypoxia induces maximal apoptosis and cell viability reductions, (Figs. 1B and C) and is associated with maximal LC3-II accumulation (Figs. 2A and B), a possible causal link is suggested.

### Evidence for a causal relationship between combination treatment induced autophagic vacuole accumulation and tumor cell death

**Figure 3:**
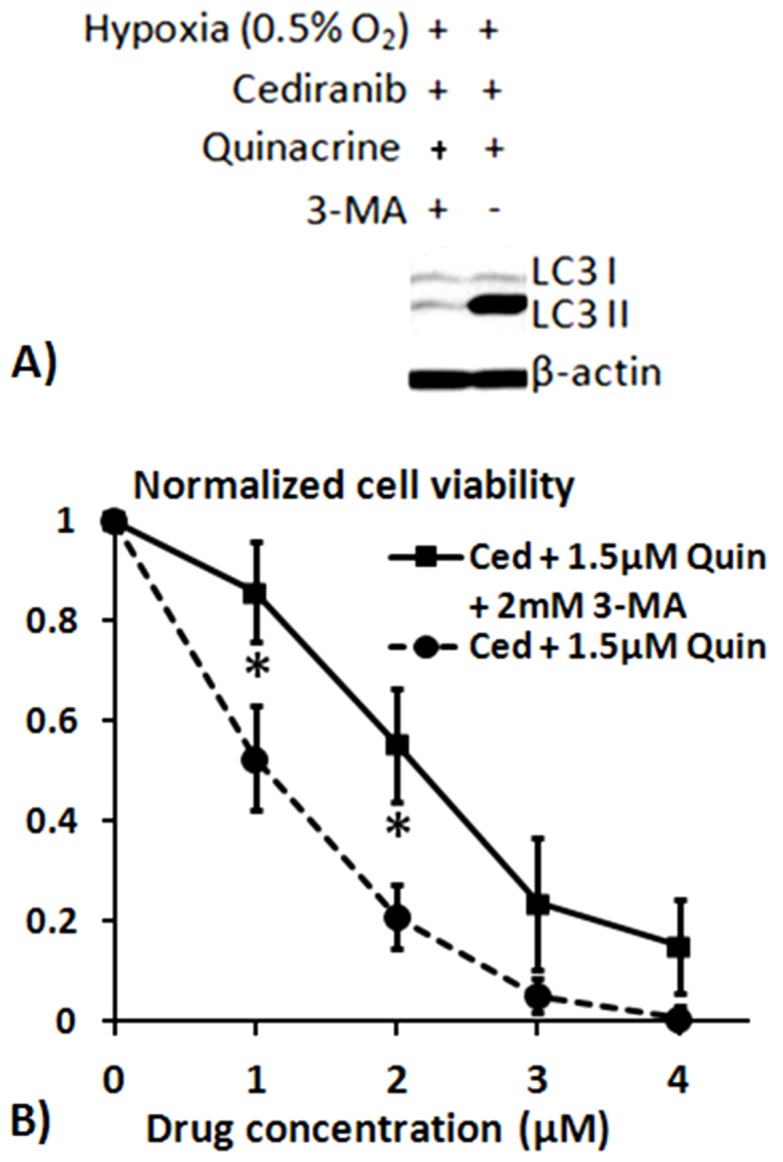
3-MA prevents LC3 accumulation and reduces cediranib/quinacrine efficacy. A) Representative LC3 western blots obtained from 4C8 cells grown under hypoxic (0.5% O_2_) conditions for 24 hrs while exposed to a combination of cediranib (2 µM) and quinacrine (1.5 µM), either with or without 2 mM 3-MA. B) Results from MTS cell viability assays showing mean 4C8 cell viability in response to treatment, normalized to untreated samples. Assays were performed after cells were exposed to treatment for 72 hours, under hypoxic (0.5% O_2_) conditions. Results are mean ± SEM of three independent experiments, performed in triplicate. Significant differences between tumor cell viability results obtained with and without 3-MA are denoted by * (p<0.05).

We next sought to determine whether the autophagic vacuole accumulation in response to combination treatment under hypoxic conditions is not only associated with, but also plays a causal role in the anti-glioma effects. To accomplish this, we exposed 4C8 cells to combination treatment under hypoxic conditions with and without the addition of early stage autophagy inhibitor, 3-Methyladenine (3-MA). 3-MA blocks autophagosome formation via the inhibition of type III Phosphatidylinositol 3-kinases (PI-3K) [18]. In agreement with Fig. 2A, LC3-II levels indicated that there was an increase in autophagic vacuoles with the combination treatment. Notably, this increase was almost entirely inhibited in the presence of 3-MA (Fig. 3A). We next determined if the prevention of autophagic vacuole accumulation by 3-MA reduced cytotoxicity of the combination treatment. Fig. 3B shows that there was a substantial and statistically significant increase in 4C8 cell viability in the presence of 3-MA, providing strong evidence that autophagic vacuole accumulation plays an important causal role in the enhanced cytotoxic effects of the combination treatment.

### Cediranib and quinacrine both inhibit Akt kinase activation

**Figure 4:**
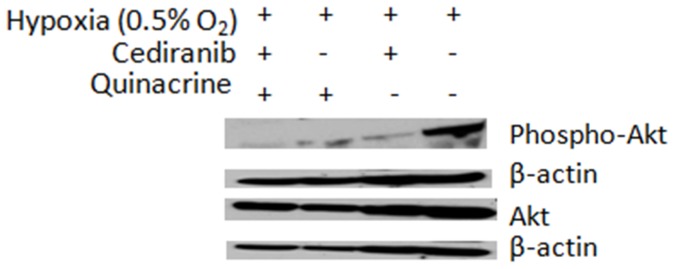
Cediranib and quinacrine inhibit Akt activation. Western blot analysis of basal Akt and phospho-Akt levels in 4C8 cultures grown under hypoxic (0.5% O_2_) conditions for 24 hrs while untreated or exposed to cediranib (2.5 µM), quinacrine (2.5 µM), or combined cediranib/quinacrine.

A key control point of autophagy is mTOR (mammalian target of rapamycin). Inhibitors of the phosphoinositide 3-kinase/Akt/mammalian target of rapamycin (PI3K/Akt/mTOR) pathway have been shown to potently induce autophagy in tumors [7], [9], [10], [11], [12], [13]. Because cediranib targets both PDGF and VEGF signaling, inhibition of which can have downstream inhibitory effects on the PI3K/Akt/mTOR pathway [3], [4], [5], [20], [31], we assessed whether cediranib treatment affected Akt kinase activation in 4C8 glioma cells. Importantly, Fig. 4 shows that cediranib strongly inhibited the phosphorylation of the Akt kinase (phospho-Akt/Akt ratio reduced from 0.57 to 0.05) suggesting a plausible mechanism by which cediranib induces autophagy. We further assessed whether quinacrine affects Akt activation. Fig. 4 indicates that similar to cediranib, quinacrine also strongly inhibits Akt activation (phospho-Akt/Akt ratio reduced to 0.09). The cediranib/quinacrine combination further reduced phospho-Akt (phospho-Akt/Akt ratio reduced to 0.02). Interestingly, this observation suggests that in addition to inhibiting autophagic vacuole degradation through its lysosomotropic action, quinacrine may also have the capability to modulate autophagy via other mechanisms which may further enhance its therapeutic action. A stimulation of autophagosome induction through Akt inhibition would expectedly enhance the autophagic vacuole accumulation induced by its inhibition of autophagic vacuole degradation.

### Quinacrine is more therapeutically effective than bafilomycin A1

**Figure 5:**
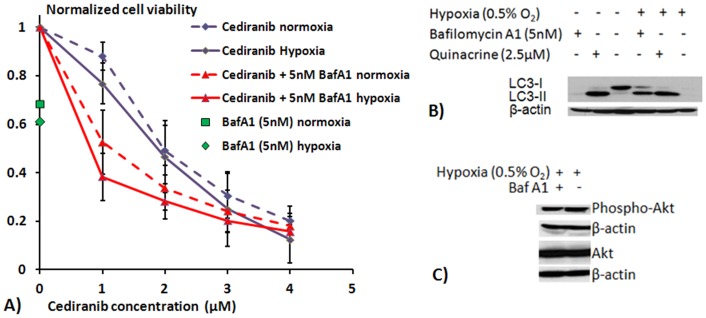
Bafilomycin A1/cediranib efficacy and bafilomycin A1 effects on LC3 and Akt. A) Results from MTS cell viability assays showing mean 4C8 cell viability in response to treatment, normalized to untreated samples. Assays were performed after cells were exposed to cediranib and/or bafilomycin A1 treatment for 72 hours, under normoxic or hypoxic (0.5% O_2_) conditions. Results are mean ± SEM of three independent experiments, performed in triplicate. B) Representative LC3 western blots obtained from 4C8 cells grown under normoxic or hypoxic (0.5% O_2_) conditions for 10 hrs while untreated or exposed to quinacrine (0.8 µM) or bafilomycin A1 (5 nM). C) Representative basal Akt and phospho-Akt western blots obtained from 4C8 cells grown under hypoxic (0.5% O_2_) conditions for 24 hrs while untreated or exposed to bafilomycin A1 (5 nM).

Because quinacrine's Akt inhibitory capability has the potential to increase its therapeutic efficacy, we compared it to bafilomycin A1, a commonly employed late stage autophagy inhibitor. Bafilomycin A1 is a specific inhibitor of the H^+^ ATPase (V-ATPase) enzyme, which drives acidification in lysosomes, thereby inhibiting the autophagic vacuole degradation process. [32] Fig. 5A indicates MTS cell viability assays demonstrating that single agent treatment with 5 nM bafilomycin A1 reduced 4C8 glioma cell viability by 32% under normoxic conditions. Consistent with the effects of bafilomycin A1 and hypoxia on autophagy, the cytotoxic effect of bafilomycin A1 tended to increase with hypoxia, to a 39% decrease in cell viability. However, a comparison with the combination studies in Fig. 1B, which employed quinacrine doses that produced comparable effects under normoxic conditions, shows that the enhancement under hypoxic conditions with bafilomycin A1 was clearly of a lesser magnitude than that observed with quinacrine. Fig. 5A also indicates the dose response to cediranib and cediranib in combination with 5 ηM bafilomycin A1, a concentration with a comparable effect on cell viability to the 2.5 µM quinacrine used in the combination studies in Fig. 1B. Similar to what was observed with combined cediranib/quinacrine, the combined cediranib/bafilomycin A1 exhibited an approximately additive effect under normoxic conditions, consistent with the concept that inhibition of autophagy enhances cediranib's efficacy. However, in contrast to what was observed with combined cediranib/quinacrine, efficacy was only modestly increased by hypoxia with combined cediranib/bafilomycin A1. Hence, while efficacy with combined cediranib/bafilomycyin A1 was clearly increased over cediranib alone, the data indicate that the extent of increased efficacy differs from that observed with combined quinacrine, particularly under hypoxic conditions. In order to investigate possible underlying mechanisms, we exposed 4C8 cells to the same doses of bafilomycin A1 and quinacrine that were used for the cediranib combination studies and assessed LC3-II accumulation. Importantly, we observed (Fig. 5B) that under both normoxic and hypoxic conditions, quinacrine induced a larger accumulation of LC3-II than bafilomycin A1. Further experiments probed the levels of phosphorylated Akt kinase in response to bafilomycin A1 treatment and found that, in contrast to quinacrine, there was no change in the activation of Akt kinase (Fig. 5C). This finding is consistent with the concept that quinacrine may induce increased efficacy in comparison to bafilomycin A1, by not only inhibiting the degradation of autophagic vacuoles, but also by driving increased autophagosome induction through its inhibitory effects on Akt. Hence, it may act in concert with cediranib in this manner, which also targets Akt.

## Discussion

RTK signaling plays a critical role in tumorigenic processes, driving proliferation, survival, and angiogenesis through modulation of key downstream pathways such as PI3K/Akt/mTOR, Ras/Raf/MEK/MAPK, and others [3], [4], [5]. The use of specific RTK inhibitors could potentially provide a more targeted anticancer therapeutic approach with lesser nonspecific toxicity than conventional chemotherapy. Recent years have seen FDA approval of several RTK inhibitors, including sunitinib (targets include VEGFR and PDGFR) for the treatment of gastrointestinal stromal tumor and sorafenib (targets include Raf, PDGFR, VEGFR) for the treatment of advanced renal cell carcinoma and other tumor types. As with cediranib, these particular agents have both anti-tumor and anti-angiogenic capability. Cediranib and other RTK inhibitors however, have generally not yet demonstrated single agent clinical efficacy with malignant glioma [3], [4], [5], [33], [34], [35]. Various resistance mechanisms can occur in response to molecularly targeted therapeutics. The redundancy of intracellular signaling pathways, loss of negative inhibition, mutations leading to constitutive activation, as well tumor heterogeneity all can play a role [3], [4], [5]. Tumors can also become resistant over time through acquired secondary mutations within the targeting agent's binding site. For malignant glioma, the targeting of infiltrating cells in brain regions maintaining a relatively intact blood brain barrier (BBB) may be of limited success because the agent either fails to cross the BBB, or is removed via active efflux mechanisms. Various resistance mechanisms can also counteract the anti-angiogenic efficacy of targeted agents, by activating alternative pro-angiogenic signaling pathways, or activating invasion and metastasis to access normal tissue vasculature [6], [34]. Furthermore, various mechanisms can induce adaptation to the hypoxic stress induced by anti-angiogenic agents [10], [25], [36]. Autophagy is known to be a key component of the hypoxic stress response. Autophagy occurs at basal levels in most cells and aids in the routine turnover of cytoplasmic constituents. Under hypoxic/nutrient stress conditions however, autophagic flux can increase in tumor cells and play a cytoprotective role by removing damaged, toxic proteins and organelles and providing substrates for energy metabolism and the biosynthetic needs of the cell. The HIF-1 transcription factor is activated in response to intratumoral hypoxia and upregulates autophagy, in addition to other cellular processes which enable adaptation to hypoxic stress [10], [25], [37], [38], [39] [10], [25], [37], [38], [39]. Another key player is the unfolded protein response (UPR), activated in response to endoplasmic reticulum (ER) stress, resulting from the accumulation of toxic misfolded proteins, a condition exacerbated by hypoxia. [40] The UPR slows protein synthesis, upregulates molecular chaperones, and upregulates degradation of misfolded proteins, in part by strongly inducing autophagy. [40], [41] Additionally AMP-activated protein kinase (AMPK) is a crucial cellular energy sensor which activates autophagy under hypoxic conditions, via effects on mTOR. In addition to conferring cytoprotection under hypoxic/nutrient stress conditions, autophagy is increasingly being demonstrated to play a role in drug resistance, and is known to be activated in response to commonly used therapeutic agents such as temozolomide, bevacizumab and others. [8], [9], [11], [12], [13], [14], [15], [16], [42] Additionally, while autophagy contributes to cell survival, it is also involved in cell death regulation. It has been shown that a late stage inhibition of autophagy, targeting autophagic vacuole degradation, can trigger cell death. [9], [11], [12], [13], [14], [15], [16], [17], [18] Although the precise mechanism of autophagic cell death is not known, an accumulation of autophagic vacuoles has been associated with a disruption in mitochondrial and lysosomal membrane permeability, leading to tumor cell death through a process that may have significant crosstalk with the apoptotic pathway. [9], [18], [43], [44] Thus, when autophagic flux has been sufficiently upregulated in tumor cells, they can be very sensitive to lysosomotropic agents such as chloroquine and quinacrine through their late stage inhibition of the process. Although chloroquine is more widely used in cancer research, quinacrine has better blood brain barrier penetration and has been shown to exert more robust cytotoxicity than chloroquine. [18], [45]

Employing Western blot and fluorescent microscopy to assess both the levels and spatial distribution of autophagic vacuole biomarker LC3, our study determined that, consistent with our previous findings, treatment with cediranib, quinacrine, or hypoxia each induced autophagic vacuole accumulation in glioma cells. [19] The combined treatment under hypoxic conditions drove autophagic vacuole accumulation to maximal levels, and notably, also induced maximal apoptosis (Fig. 1C). These observations are consistent with inhibition of autophagic vacuole degradation by quinacrine, occurring in combination with a stimulation of autophagosome induction. To assess the causality of autophagic vacuole accumulation in therapeutic efficacy we employed the early stage autophagy inhibitor 3-MA, and determined that with combined cediranib/quinacrine under hypoxic conditions, 3-MA administration both completely blocked autophagic vacuole accumulation and markedly attenuated cytotoxicity. The findings provide key evidence that that induction of autophagic vacuole accumulation is not only associated with the cytotoxicity of the drug combination, but is likely to play a causal role. The observations support the hypothesis that the profound *in vivo* effects that we reported previously in intracranial 4C8 glioma with combined cediranib/quinacrine treatment relates at least partly to an induction of autophagic cell death. The findings also support the concept that the *in vivo* efficacy with the combined agents could substantially derive from their direct effects on glioma cells. However, because cediranib's molecular targets are also potently anti-angiogenic, a similar efficacy against endothelial cells cannot be excluded, consistent with the marked tumor devascularization that was observed. Various reports have indicated a susceptibility of proliferating endothelial cells to autophagic cell death and/or autophagy inhibition. [46], [47], [48]

Consistent with the known stimulation of autophagy by hypoxia, our study has clearly identified an important role for hypoxia in enhancing the synergistic efficacy of the cediranib/quinacrine combination in 4C8 glioma. Importantly, both the reduction of cell viability and the induction of apoptosis induced by the therapeutic combination was markedly potentiated by hypoxia (Fig. 1B-C) and was observed in conjunction with a marked accumulation of autophagic vacuoles (Fig. 2A-B). Our results also indicated that autophagic vacuole accumulation plays a key role in the combined cediranib/quinacrine efficacy. Hence, taken together, the data strongly suggest that that hypoxia potentiates efficacy through its stimulation of the autophagic process. These findings underscore the importance of hypoxia as a key tumor microenvironment variable which can affect therapeutic outcome. Our study, employing EF5 immunostaining, documents that hypoxia is prevalent even in untreated 4C8 intracranial glioma (Fig. 1A). This observation further supports a role for hypoxia in our previous *in vivo* observations of combined cediranib/quinacrine efficacy in 4C8 glioma. [19] Notably, the perfusion MRI results in that study also indicated that cediranib's anti-angiogenic effects are likely to further exacerbate hypoxia in the *in vivo* setting, via cediranib-induced reductions of blood flow within the tumor core region. [19] The observations suggest that cediranib is likely to promote autophagic flux in tumors both by modulating cell signaling and by increasing hypoxic stress via its anti-angiogenic effects. Our determination that cediranib/quinacrine treatment efficacy is strongly interactive with the hypoxic condition is of key importance in regards to treatment of tumors, which often are substantially hypoxic. [10], [23], [25], [36] This is particularly the case with malignant glioma, a disease characterized by focal hypoxic regions with pseudopalisading necrosis. [1], [24], [25] The radiation treatment resistance of hypoxic tumor regions is well established, and constitutes a limitation for a key component of the standard glioma treatment. [49] Secondly, tumor cells are very adaptable to hypoxia, in part through autophagy and other mechanisms, and it has been well documented that hypoxia effectively promotes an aggressive tumor phenotype, with upregulation of glycolytic metabolism, angiogenesis, proliferation and survival. [10], [23], [25], [36] The enhanced efficacy of combined cediranib/quinacrine treatment under hypoxic conditions would suggest that it could offer a critical advantage in targeting tumor hypoxic regions. Our demonstration of the prevalence of hypoxic regions in intracranial 4C8 glioma, in tandem with our recently demonstrated *in vivo* success with combined cediranib/quinacrine treatment in that model, is consistent with this premise.

In our study, we determined that autophagic pathway stimulation not only occurs with hypoxia, but also as a direct result of cediranib treatment. Such an effect is likely to occur via inhibition of one or more of cediranib's RTK targets, which can activate various important downstream signaling pathways, such as PI3K/Akt/mTOR, inhibition of which would stimulate autophagic flux through downregulation of mTOR, the primary autophagy control point. [4], [5] Cediranib potently inhibits multiple RTKs, including vascular endothelial growth factor (VEGF) receptors 1, 2, and 3, platelet derived growth factor (PDGF) receptors (a and b), and c-Kit [20], A recent report indicates that Cediranib also effectively targets EGFR, MET, and other RTK receptors in glioma, and effectively inhibits Akt in U251 glioma. [50] Consistent with this, we determined that cediranib strongly inhibits Akt signaling in 4C8 glioma. Akt is one of the primary signaling inputs of mTOR, and hence the data provide compelling evidence that cediranib stimulates autophagy and autophagic vacuole accumulation at least partly via inhibition of the PI3K/Akt/mTOR pathway. Future studies are required to determine the primary signaling mechanisms by which cediranib inhibits Akt, PDGFR is a likely candidate for involvement, as PDGF-α receptors are well expressed on glioma cells, and studies have demonstrated PDGF-B signaling to be an important oncogenic signaling pathway in glioma. [51], [52]

While quinacrine's lysosomotropic action is well documented and required for its anti-malarial capability, it has pleiotropic biological effects. [18], [45], [53], [54] Our study reveals compelling evidence that its modulation of autophagy and therapeutic efficacy is likely to involve more than inhibition of autophagic vacuole degradation. Notably, our data clearly demonstrates that, like cediranib, quinacrine strongly inhibits Akt signaling in 4C8 glioma (Fig. 4), consistent with recent reports regarding quinacrine and the related compound 9-aminoacridine in other tumor cell types. [54], [55] We observed maximal Akt inhibition with combined cediranib/quinacrine treatment. Integrated with quinacrine's ability to inhibit autophagic vacuole degradation, this observation has important therapeutic implications, suggesting that quinacrine, through inhibition of Akt/mTOR signaling, could both stimulate autophagosome induction while simultaneously inhibiting autophagic vacuole degradation, thereby exacerbating autophagic vacuole accumulation. Our experiments with bafilomycin A1 support this therapeutic mechanism. Bafilomycin A1 interrupts autophagic vacuole degradation through specific inhibition of the vacuolar H^+^ ATPase. Importantly, our data clearly demonstrates that unlike quinacrine, it does not inhibit Akt phosphorylation (Fig. 4). Notably, we observed markedly increased autophagic vacuole accumulation with quinacrine in comparison to bafilomycin A1 under both normal and hypoxic conditions. The difference was particularly pronounced under normal oxygen conditions, when autophagic stimulation is generally at basal levels. Bafilomycin A1 induced minimal autophagic vacuole accumulation under normoxic conditions, while in contrast, a pronounced accumulation was observed with quinacrine, consistent with a stimulation of autophagosome induction. Importantly, when in combination with cediranib, increased therapeutic efficacy was observed with quinacrine over bafilomycin A1 under hypoxic conditions. This observation is consistent with the concept that the inhibitory convergence on Akt/mTOR signaling by cediranib/quinacrine promotes increased cytotoxic autophagic vacuole accumulation. Additionally, as Akt signaling is known to be a primary driver of growth and survival in glioma, its effective targeting is likely to involve multiple therapeutic mechanisms. [3], [4], [5], [56] Hence, our data suggests quinacrine to be a unique therapeutic agent which targets a key oncogenic signaling pathway and modulates autophagy in a complex manner, properties which can be exploited to substantially enhance the efficacy of the RTK inhibitor cediranib.

In conclusion, our results provide compelling evidence that the unique anti-glioma cytotoxic efficacy of combined cediranib/quinacrine is causally linked to modulations in the autophagic pathway by the two agents, and by hypoxia. Our study provides a basis for an examination of this combination therapy in future clinical trials.
